# Lipophosphoglycans from *Leishmania amazonensis* Strains Display Immunomodulatory Properties via TLR4 and Do Not Affect Sand Fly Infection

**DOI:** 10.1371/journal.pntd.0004848

**Published:** 2016-08-10

**Authors:** Paula M. Nogueira, Rafael R. Assis, Ana C. Torrecilhas, Elvira M. Saraiva, Natália L. Pessoa, Marco A. Campos, Eric F. Marialva, Cláudia M. Ríos-Velasquez, Felipe A. Pessoa, Nágila F. Secundino, Jerônimo N. Rugani, Elsa Nieves, Salvatore J. Turco, Maria N. Melo, Rodrigo P. Soares

**Affiliations:** 1 Centro de Pesquisas René Rachou/FIOCRUZ, Belo Horizonte, Minas Gerais, Brazil; 2 Departamento de Parasitologia, UFMG, Belo Horizonte, Minas Gerais, Brazil; 3 Laboratório de Imunologia Celular e Bioquímica de Fungos e Protozoários, Departamento de Ciências Biológicas, UNIFESP, São Paulo, São Paulo, Brazil; 4 Laboratório de Imunobiologia das Leishmanioses, Departamento de Imunologia, UFRJ, Rio de Janeiro, Rio de Janeiro, Brazil; 5 Centro de Pesquisas Leônidas e Maria Deane/FIOCRUZ, Manaus, Amazonas, Brazil; 6 Laboratório de Parasitologia Experimental, Departamento de Biologia, Universidad de Los Andes, Mérida, Venezuela; 7 Department of Biochemistry, University of Kentucky Medical Center, Lexington, Kentucky, United States of America; Liverpool School of Tropical Medicine, UNITED KINGDOM

## Abstract

The immunomodulatory properties of lipophosphoglycans (LPG) from New World species of *Leishmania* have been assessed in *Leishmania infantum* and *Leishmania braziliensis*, the causative agents of visceral and cutaneous leishmaniasis, respectively. This glycoconjugate is highly polymorphic among species with variation in sugars that branch off the conserved Gal(β1,4)Man(α1)-PO_4_ backbone of repeat units. Here, the immunomodulatory activity of LPGs from *Leishmania amazonensis*, the causative agent of diffuse cutaneous leishmaniasis, was evaluated in two strains from Brazil. One strain (PH8) was originally isolated from the sand fly and the other (Josefa) was isolated from a human case. The ability of purified LPGs from both strains was investigated during *in vitro* interaction with peritoneal murine macrophages and CHO cells and *in vivo* infection with *Lutzomyia migonei*. In peritoneal murine macrophages, the LPGs from both strains activated TLR4. Both LPGs equally activate MAPKs and the NF-κB inhibitor p-IκBα, but were not able to translocate NF-κB. *In vivo* experiments with sand flies showed that both stains were able to sustain infection in *L*. *migonei*. A preliminary biochemical analysis indicates intraspecies variation in the LPG sugar moieties. However, they did not result in different activation profiles of the innate immune system. Also those polymorphisms did not affect infectivity to the sand fly.

## Author Summary

*Leishmania amazonensis*, a member of the *Leishmania mexicana* complex, is the causative agent of localized cutaneous leishmaniasis (LCL) and anergic diffuse cutaneous leishmaniasis (ADCL) [1,2]. It is widely distributed throughout the Amazon basin, where it infects a wide range of terrestrial rodents and, less frequently, marsupials. Its main vector is *Lutzomyia flaviscutellata* (Diptera: Psychodidae) widely distributed in South America and a recent study has predicted its expansion towards South of Brazil [3]. Moreover, *Lutzomyia migonei* (França, 1920) can also harbor the infection of this species [4,5]. Although its transmission to man is very uncommon, *L*. *amazonensis* triggers an incurable and disseminated form of cutaneous leishmaniasis [2,6]. However, most of the mechanisms involved in *L*. *amazonensis* pathogenesis are still unknown, especially those related to surface molecules. Glycoconjugates have been extensively characterized as important for the establishment of infection as they protect the parasite from the early action of the host immune system and therefore acting as invasive/evasive strategies. Consequently, we here present the role of lipophosphoglycan (LPG) of *L*. *amazonensis* in the interaction with vertebrate and invertebrate hosts.

## Introduction

The major cell surface glycoconjugate of *Leishmania* is the lipophosphoglycan (LPG), implicated in a wide range of functions, both in vertebrate and invertebrate hosts [7]. In the invertebrate host, LPG variations are important for *Leishmania* specificity to the sand fly [8], where attachment of the parasite to a midgut receptor is a crucial event [9]. In the vertebrate host, the main functions of this virulence factor during the earlier steps of infection include: protect the parasite from complement-mediated lysis, attachment and entry into macrophages [10], able to inhibit phagolysosomal fusion [11], modulation of nitric oxide (NO) production [12] and inhibition of protein kinase C (PKC) [13]. Interestingly, although *L*. *major* LPG mutants (*lpg1*^*-*^) were highly susceptible to complement mediated lysis, they were able to invade macrophages reinforcing the role of other molecules and the host defenses during the interaction [11].

Many functions have been attributed to *L*. *amazonensis* LPG including induction of neutrophil extracellular traps (NETs) [14], induction of protein kinase R (PKR) [15], triggering and killing of the parasite via Leukotriene B4 (LTB4) [16]. Although *L*. *amazonensis* LPG is important in many steps of host infection, its role during the interaction with macrophages and sand flies remains unknown.

LPG structures have been described for several dermotropic and viscerotropic *Leishmania* [17–26]. LPGs have a conserved glycan core region of Gal(α1,6)Gal(α1,3)Gal_f_(β1,3)[Glc(α1)-PO_4_]Man(α1,3)Man(α1,4)-GlcN(α1) linked to a 1-*O*-alkyl-2-*lyso*-phosphatidylinositol anchor. The salient feature of LPG is another conserved domain consisting of the Gal(β1,4)Man(α1)-PO_4_ backbone of repeat units (*n =* ~15–30). The distinguishing feature of LPGs that is responsible for the polymorphisms among *Leishmania* spp. is variable sugar composition and sequence of branching sugars attached to the repeat units and cap structure [27]. For example, the LPG of *Leishmania major* (Friedlin) has β-1,3 galactosyl side-chains, often terminated with arabinose, whereas the LPGs of *Leishmania donovani* (Mongi) and *L*. *infantum* (PP75 and BH46 strains) possess β-glucoses in their repeat units [17,20,24]. However, there is no available information on the degree of variability in the LPG structure for *L*. *amazonensis*.

The *L*. *major* LPG was identified as potent agonist of Toll-like receptor 2 (TLR2) in human natural killer (NK) cells and murine macrophages, triggering the production of TNF-α and IFN-γ through MyD88 [28,29]. Recently, the LPGs of two New World species (*L*. *infantum* and *Leishmania braziliensis*) differentially activated TLR2. In this case, *L*. *braziliensis* LPG was more pro-inflammatory being able to induce the translocation of NF-κB to the nucleus [30].

As a part of a wider project on the glycobiology of New World species of *Leishmania*, we evaluated the role of *L*. *amazonensis* LPGs (PH8 and Josefa strains) during the interaction with host cells and the sand fly *L*. *migonei*. The present study might help to improve our understanding on the immune modulation mediated by glycoconjugates of *L*. *amazonensis*, the etiological agent of diffuse cutaneous leishmaniasis (DCL).

## Materials and Methods

### Ethics statement

The animals were kept in the Animal Facility of the Centro de Pesquisas René Rachou/FIOCRUZ. All animals were handled in strict accordance with animal practice as defined by Internal Ethics Committee in Animal Experimentation (CEUA) of Fundação Oswaldo Cruz (FIOCRUZ), Belo Horizonte, Minas Gerais (MG), Brazil (Protocol P-82/11-4). This protocol followed the guidelines of CONCEA/MCT, the maximum ethics committee of Brazil. Knockout mice handling protocol was approved by the National Commission of Biosafety (CTNBio) (protocol #01200.006193/2001-16).

### Parasites, growth curves, and molecular typing

World Health Organization Reference strains of *L*. *amazonensis* (IFLA/BR/1967/PH8 and MHOM/BR/75/Josefa) were used. The PH8 strain was originally isolated from the sand fly *L*. *flaviscutellata* from Pará State, Brazil, and the Josefa strain was isolated from a human case from Bahia State, Brazil. Promastigotes were cultured in M199 medium supplemented with 10% fetal bovine serum (FBS), penicillin 100 units/mL, streptomycin 50 μg/mL, 12.5 mM glutamine, 0.1 M adenine, 0.0005% hemin, and 40 mM Hepes, pH 7.4 at 26°C until late log phase [21]. Parasites were seeded in triplicate (1 x 10^5^ cells/mL), and growth curves of PH8 and Josefa strains were determined daily using a Neubauer improved haemocytometer until cells reached a stationary phase. Both strains exhibited a similar division profile reaching stationary phase after 7 days of culture. For this reason the 6^th^ day was chosen for harvesting parasites for LPG extraction and molecular typing (S1A Fig).

For molecular typing, genomic DNA was extracted from log-phase *Leishmania* using the phenol/chloroform method (1:1) for amplification of the HSP70 fragment prior to digestion with HaeIII as previously described [31]. Positive controls included DNA from *L*. *braziliensis* (MHOM/BR/75/M2903), *L*. *infantum* (MHOM/BR/74/PP75), *Leishmania guyanensis* (MHOM/BR/75/M4147) and *L*. *amazonensis* (IFLA/BR/67/PH8). After PCR-RFLP both *L*. *amazonensis* strains were confirmed (S1B Fig).

### Extraction and purification of LPG

**Fig 1:**
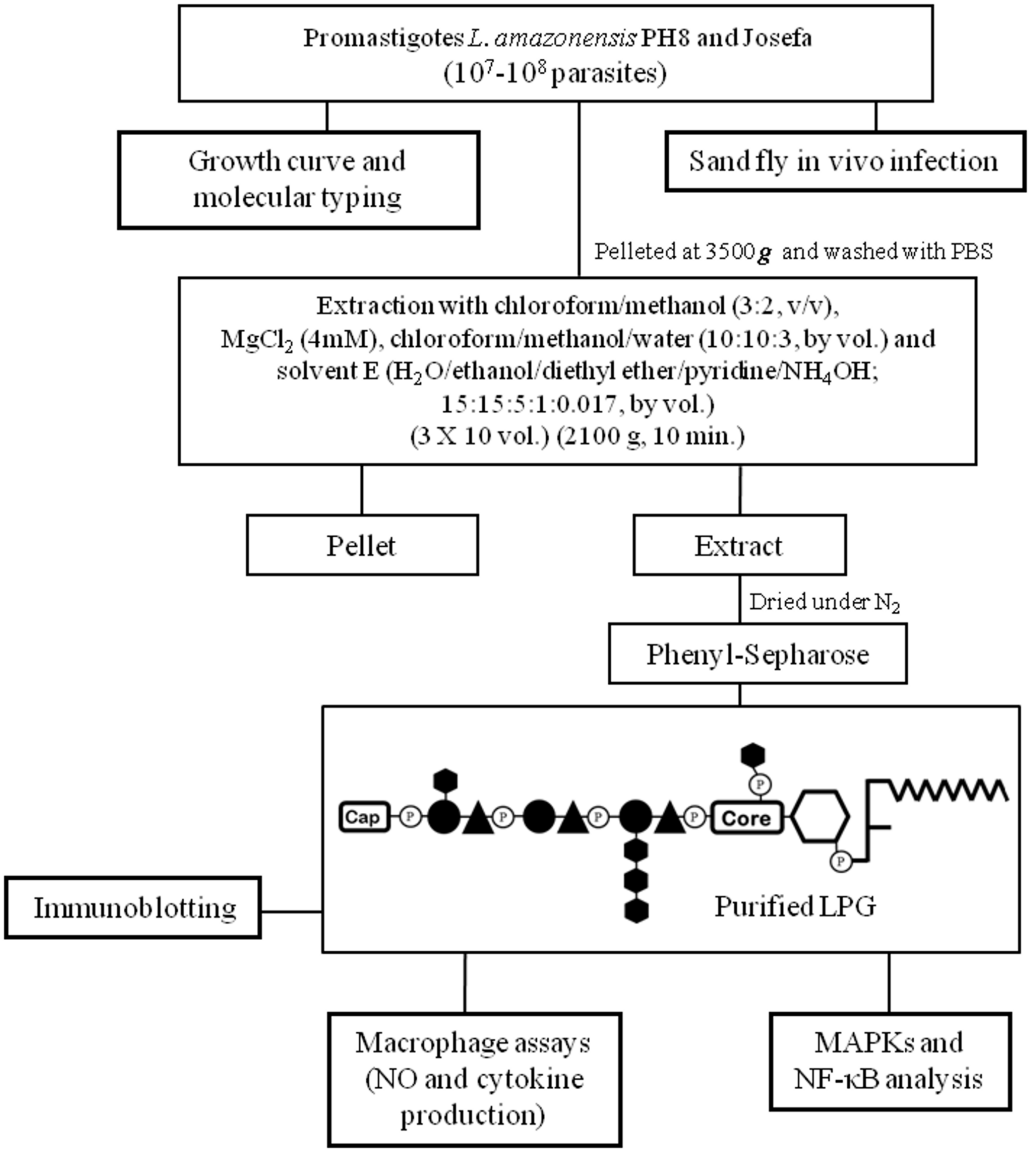
Procedures for extraction, purification, preliminary characterization of *L*. *amazonensis* LPG, interaction with vertebrate cells and *L*. *migonei*. Late log phase cells were harvested and washed with PBS. For studies with vector, *L*. *migonei* midguts were dissected on days 2 and 4 post feeding containing *L*. *amazonensis* from each strain. Parasite cell pellets were subject to extraction with organic solvents as described elsewhere. For purification, the solvent E extract was dried under N_2_ evaporation and applied into a phenyl-Sepharose column. The purified LPG was used for biological and immunological assays.

For optimal LPG extraction, late log phase cells were harvested and washed twice with PBS prior to extraction of LPGs (Fig 1). The LPG extraction was performed as described elsewhere with solvent E (H_2_O/ethanol/diethylether/pyridine/NH_4_OH; 15:15:5:1:0.017) after a sequential organic solvent extraction [32]. For purification, the solvent E extract was dried under N_2_ evaporation, resuspended in 2 mL of 0.1 M acetic acid/0.1 M NaCl, and applied onto a column with 2 mL of phenyl-Sepharose, equilibrated in the same buffer. The column was washed with 6 mL of 0.1 M acetic acid/0.1 M NaCl, then 1 mL of 0.1 M acetic acid and finally 1 mL of endotoxin free water. The LPGs were eluted with 4 mL of solvent E then dried under N_2_ evaporation. LPG concentrations were determined as described elsewhere [33]. Prior to use on *in vitro* cells cultures, LPGs were diluted in RPMI. All solutions were prepared in sterile, LPS-free distilled water (Sanobiol, Campinas, Brazil). All extractions and purifications procedures are depicted in Fig 1.

### Immunoblotting and preliminary characterization of LPGs

Purified LPGs (5 μg) were subjected to dot-blot, blocked (1 h) in 5% milk in PBS and probed for 1 h with monoclonal antibody (mAb) CA7AE (1:1000), that recognizes the unsubstituted Gal(β1,4)Man repeat units [34]; mAb LT22 (1:1000) that recognizes β-glucose side chains and WIC 79.3 (1:1000) that recognizes β-galactose side chains [21,35]. After three washes in PBS (5 min), the membrane was incubated for 1 h with anti-mouse IgG conjugated with peroxidase (1:5,000) and the reaction was visualized using luminol.

### Purification of murine peritoneal macrophages and cell culture

Thioglycollate-elicited macrophages were extracted from C57BL/6 and C57BL/6 knockouts TLR2 (-/-) and TLR4 (-/-) by peritoneal washing with ice cold RPMI and enriched by plastic adherence (1 h, 37°C, 5% CO_2_). Cells (3 x 10^5^ cells/well) were washed with fresh RPMI then culture in RPMI, 2 mM glutamine, 50 U/mL of penicillin and 50 μg/mL streptomycin supplemented with 10% FBS in 96-well culture plates (37°C, 5% CO_2_). Cells were primed with interferon-gamma (IFN-γ) (3 IU/mL) for 18 h prior to incubation with LPGs from both strains (10 μg/mL), live stationary *Leishmania* parasites (MOI 10:1) and lipopolysaccharide (LPS: 100 ng/mL) [30,36].

### Cytokine and nitrite measurements

For CBA multiplex cytokine detection, cells were plated, primed as describe above and incubated with LPGs and live stationary promastigotes (MOI 10:1) for 48 h. LPS was added as a positive control and medium as negative control. Supernatants were collected and IL-1β, IL-6, IL-10, IL-12p40 and TNF-α were determined using BD CBA Mouse Cytokine assay kits according to the manufacturer’s specifications (BD Biosciences, CA, USA). Flow cytometry measurements were performed on a FACSCalibur flow cytometry (BD Bioscience, Mountain View, CA, USA). Cell-QuestTM software package provided by the manufacturer was used for data acquisition and the FlowJo software 7.6.4 (Tree Star Inc., Ashland, OR, USA) was used for data analysis. A total 1,500 events were acquired for each preparation. Results are representative of six experiments in duplicate. Nitrite concentrations were determinate by Griess reaction (Griess Reagent System, 2009).

### MAPKs and NF-κB translocation assay

For MAPKs, peritoneal murine macrophages were obtained as described above. They were applied on 24 wells tissue culture plates (10^6^ cells/well) for 18 h prior to assay. The cells were washed with warm RPMI and incubated with LPG from both species for different times (5, 15, 30, 45 and 60 min) or with medium (negative control) or *E*. *coli* extracts (100 ng/mL, only 45 minutes) as positive control. p-p38, p-JNK, p-IκBα and total p38 were assayed as previously described [25]. p-IκBα antibody was provided by Dr. L. P. de Sousa. NF-κB translocation using CHO reporter lines (a kind gift by M. A. Campos) was determined as described elsewhere [30]. CHO reporter cells were plated (1 x 10^5^ cells/well) in 24-well tissue culture dishes and the LPG (0.02 and 0.2 μg/mL) from both strains was added in a total volume of 0.25 mL medium/well. The cells were examined by flow cytometry (BD Biosciences, CA, USA) and the analyses were performed using CellQuestTM software.

### Sand fly *in vivo* infection

*Lutzomyia migonei* (Baturite strain) sand flies were kept under laboratory conditions and were fed on 30% sucrose solution for 3–4 days prior to experiments. The insects were artificially fed using a chick skin membrane in a glass-feeder device. The chick skin membrane was provided by the Animal Facility of Centro de Pesquisas René Rachou/FIOCRUZ under the Protocol LW 30/10. Heparinized mouse blood (drawn intracardially from Balb/C), with penicillin (100 U/mL) and streptomycin (100 μg/mL) (37°C) containing 2 x 10^7^/mL logarithmic phase promastigotes (PH8 and Josefa strains) offered for 5 h under dark conditions [5]. Blood engorged flies were separated and maintained at 26°C with 30% sucrose. Engorged sand flies had their midguts dissected on days 2 and 4 post feeding. The midguts were homogenized in 30 μl of PBS and the number of viable promastigotes determined by counting under a Neubauer improved haemocytometer [24].

### Statistical analyses

For nitrite, cytokine measurements and *in vivo* sand fly experiments, the Shapiro Wilk test was conducted to test the null hypothesis that data were sampled from a Gaussian distribution [37]. For the non-parametric distribution, it was performed the Mann-Whitney test. Data were analyzed using GraphPad Prism 5.0 software (Graph Prism Inc., San Diego, Ca). P < 0.05 was considered significant.

## Results

### The LPGs from *L*. *amazonensis* strains display intraspecific polymorphism

The purified LPGs from *L*. *amazonensis* PH8 and Josefa strains were differentially recognized by the mAbs CA7AE and LT22 (S2 Fig). LPG from PH8 strain was recognized by CA7AE and LT22 as well as the positive control represented by *L*. *infantum* (BH46). However, a different recognition profile was observed for the Josefa strain since its LPG was weakly recognized by LT22 but not by CA7AE, indicating the presence of side-chains branching-off the repeat units. Because CA7AE recognizes Gal(β1,4)Man unsubstituted repeat units in LPG [34], these results indicate that at least some of the repeat units are indeed unsubstituted in the LPG of PH8 strain. On the other hand, the presence of side-chains suggestive of glucoses, due to LT22 reactivity, was detected in the LPGs of PH8 and Josefa strains. However, LT22 also recognized the galactose-branched repeat units of *L*. *major* (strains FV1 and LV39) indicating cross-reactivity of the antibodies, thus suggesting the presence of either glucose or galactose as side chains (S2 Fig). These data suggested an intraspecific polymorphism in the LPGs of *L*. *amazonensis* strains.

### LPGs from *L*. *amazonensis* strains equally activate NO and cytokine production via TLR4

**Fig 2:**
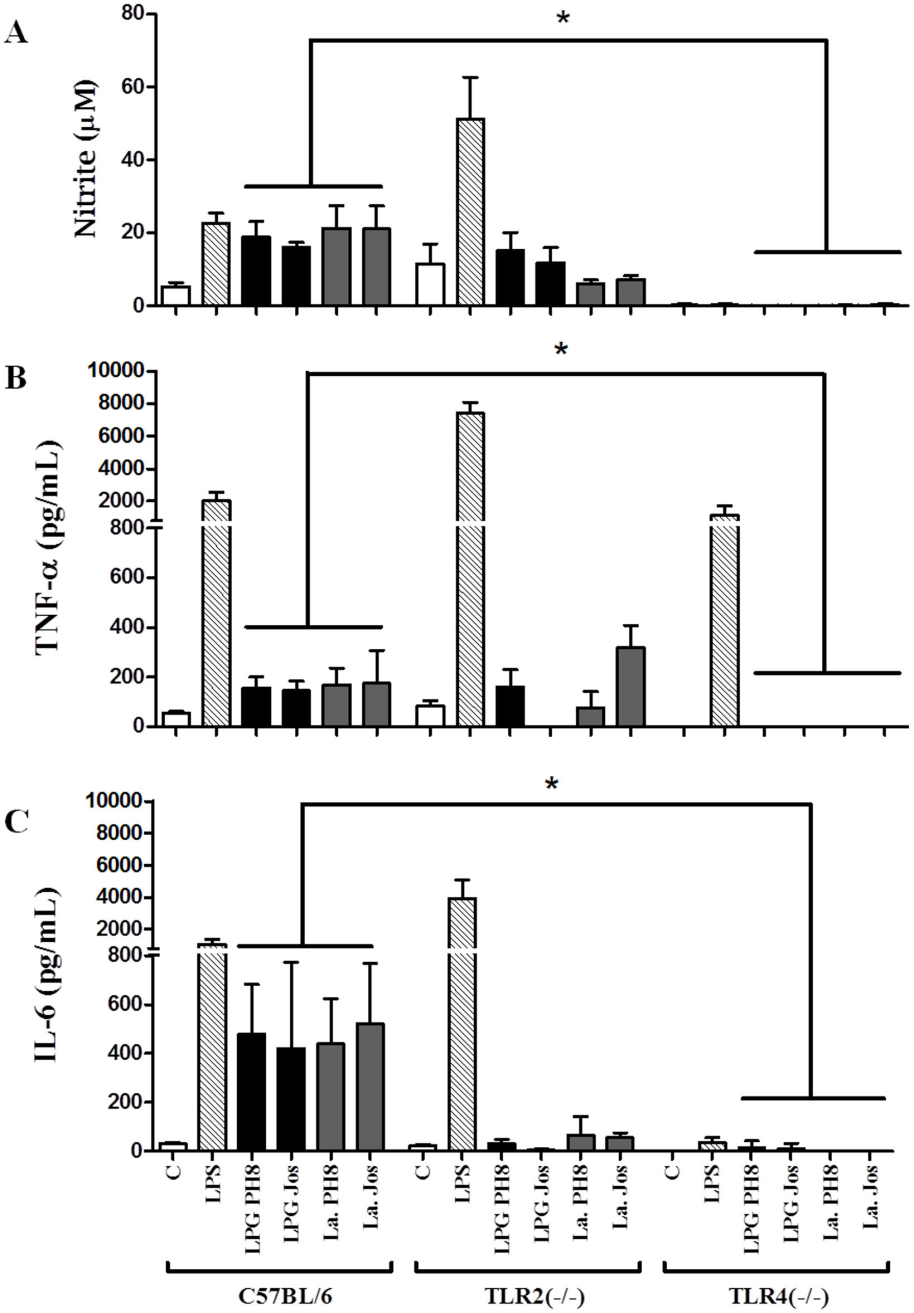
Nitrite (A) and cytokines TNF-α (B) and IL-6 (C) production by IFN-γ primed macrophages stimulated with LPG and live parasites. Cells were pre-incubated with IFN-γ (3 IU/mL) for the 18 h then 10 μg/mL of LPG, and supernatants used for cytokine and nitrite measurements were collected 48 h latter. Fresh medium alone was used as negative control cells and LPS (100 ng/mL) as a positive control. Nitrite concentration was measured by Griess reaction and cytokine concentrations were determined by flow cytometry. C = negative control; LPG PH8 = *L*. *amazonensis* LPG PH8 strain; LPG Jos = *L*. *amazonensis* LPG Josefa strain; La PH8 = *L*. *amazonensis* PH8 live promastigotes and La Jos = *L*. *amazonensis* Josefa live promastigotes. Results represent the mean ± SD of 6 experiments in duplicate, * = P< 0.05 was considered significant.

We investigated whether LPGs purified from different strains could have an impact on the parasite’s interaction with host cells, the ability to elicit NO and cytokine production by murine macrophages. LPGs from both strains were incubated with murine peritoneal macrophages from C57BL/6 and respective knockouts for TLR2 (-/-) and TLR4 (-/-). We did not detect any production of the cytokines IL-1β, IL-10 and IL-12 (S3A–S3C Fig). Both LPGs and respective parasites were able to activate through TLR4, resulting in NO, TNF-α and IL-6 production (Fig 2A–2C) (P < 0.05). As expected, LPS (positive control) activated TLR4 in the TLR2 (-/-) (Fig 2A–2C).

### LPGs from *L*. *amazonensis* equally activate MAPKs and the NF-κB inhibitor p-IκBα via TLR4

**Fig 3:**
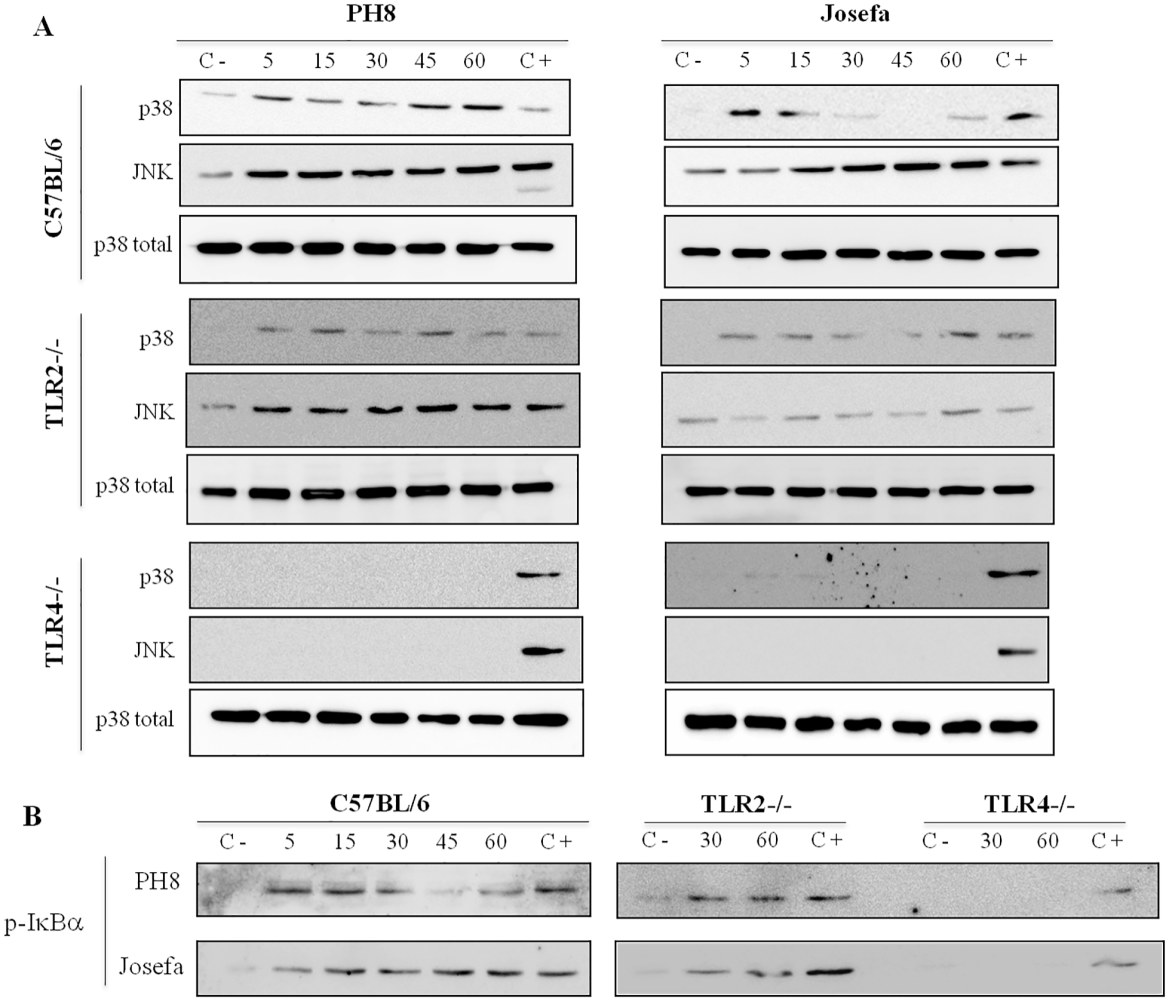
Activation of p38/JNK (A) and p-IκBα (B) in peritoneal murine macrophages (C57BL/6, TLR2 -/- and TLR4 -/-) by *L*. *amazonensis* LPGs (PH8 and Josefa). Macrophages were stimulated for 5, 15, 30, 45 and 60 min with 10 μg/mL of LPG from *L*. *amazonensis* PH8 and Josefa strains. Dually phosphorylated MAPKs (p38 and JNK) and p-IκBα were detected by Western blot analysis. C- = negative control; C+ = *E*. *coli* extract, positive control (100 ng/mL, 45 min). Total p38 content was used as the normalizing protein.

**Fig 4:**
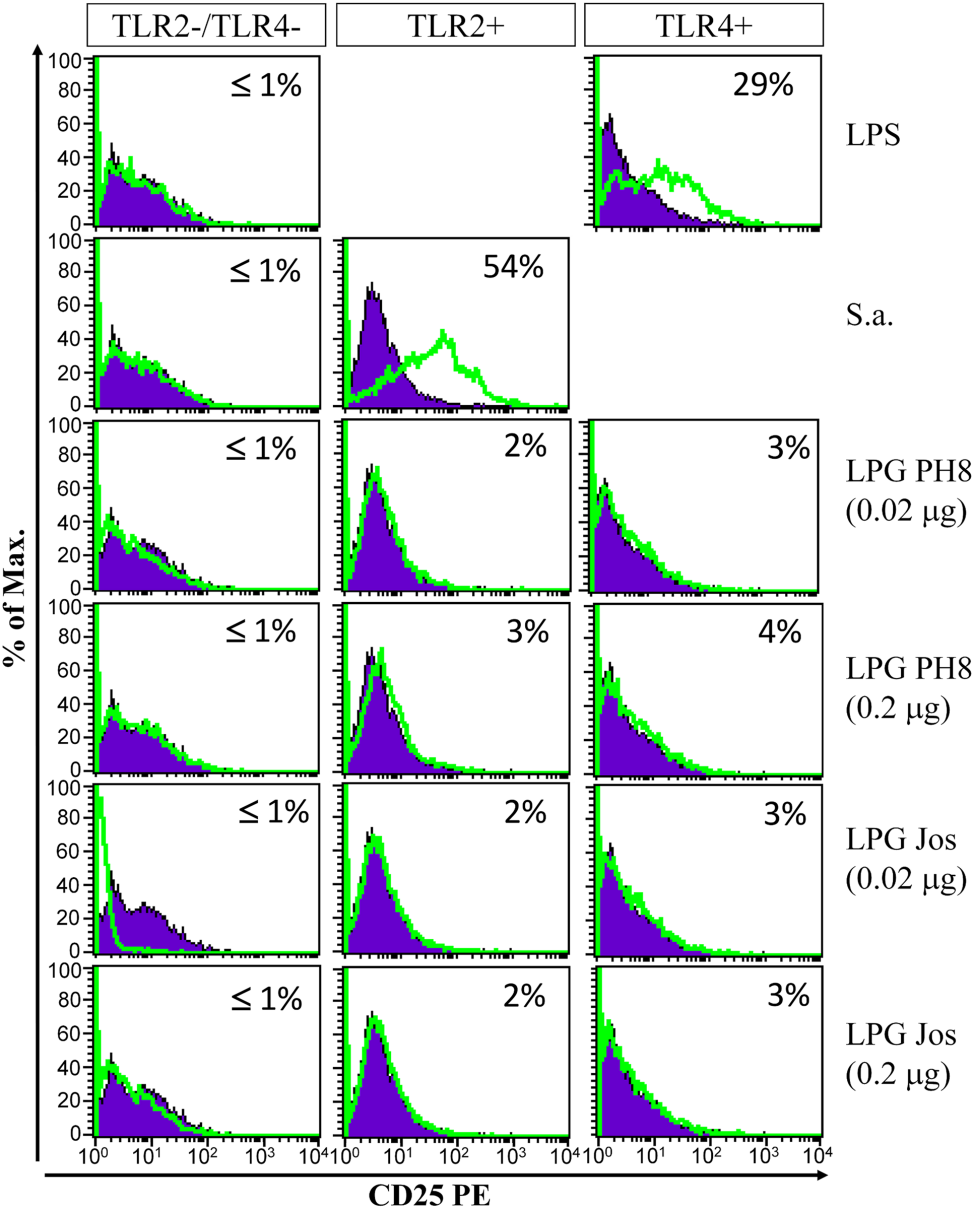
LPGs purified of *L*. *amazonensis* do not induce translocation of NF-κB through TLRs. CHO cells expressing TLR2 (TLR2+), TLR4 (TLR4+), or neither (TLR2-/TLR4-) were either untreated (purple line) or treated (green line) with LPGs from both strains of *L*. *amazonensis*. Legend: PH8 and Josefa LPGs (0.2 and 0.02 μg), Controls: LPS (TLR4 control) and *S*. *aureus* (S.a.) (TLR2 control). CD25 expression was measured by flow cytometry 18 h after stimulation. Results shown as percentage of CD25 expression on stimulated cells minus percentage of CD25 expression on non-stimulated cells.

No difference in MAPKs phosphorylation (p38 and JNK) and p-IκBα was observed after incubation with LPGs from both strains. In peritoneal murine macrophages this activation was mainly via TLR4 (Fig 3A and 3B). We also evaluated if the LPGs from these strains were able to translocate NF-κB in CHO cells. No activation of NF-κB was detected in those cells (Fig 4).

### *Leishmania amazonensis* strains equally infected the sand fly *L*. *migonei*

**Fig 5:**
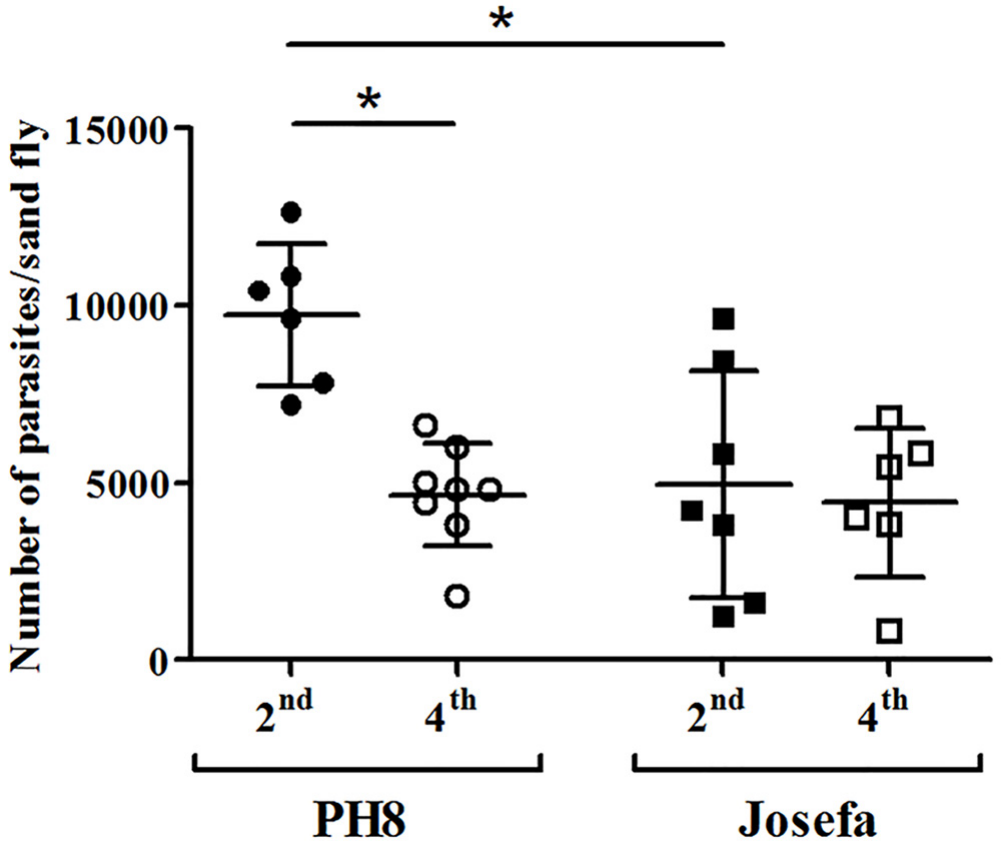
Development of *L*. *amazonensis* (PH8 and Josefa strains) in *Lutzomyia migonei*. Sand flies were infected with promastigotes (2 x 10^7^ parasites/mL) of PH8 and Josefa strains. Day 2 (2^nd^) parasites counted before blood excretion; Day 4 (4^th^) parasites remaining after blood excretion. Results are representative of two experiments and * = P < 0.05 was considered significant.

*In vivo* midgut infections of the sand flies were determined on days 2 and 4 post feeding, in order to evaluate the number of parasites after the blood meal digestion, as well as, after its excretion on day 3, where non-attached parasites are lost. Although a higher parasite density was detected for PH8 strain on day 2 (P < 0.05), no statistical differences in the parasite densities from both *L*. *amazonensis* strains were observed on day 4, and both strains were able to colonize *L*. *migonei* midgut (P > 0.05, Fig 5).

## Discussion

*Leishmania amazonensis*, etiologic agent of the cutaneous and anergic diffuse leishmaniasis, is characterized by disseminated non-ulcerative skin lesions and constantly proportion of negative delayed hypersensitivity skin-test (DTH), resulting in a high resistance of this disease to any type of chemotherapy [1,38,39]. In the Old and New World, parasite glycoconjugates have being implicated in a variety of events during parasite-host interactions [40,41]. More recently, the role of LPG and GIPLs in the *L*. *braziliensis* and *L*. *infantum* was determined, suggesting that two distinct LPGs were able to differentially modulate macrophage functions [30,41]. Regarding *L*. *mexicana* complex, from where *L*. *amazonensis* is a member, a recently study has demonstrated the inflammatory role of LPG [42]. This glycoconjugate naturally exposed to the host immune system could contribute to the maintenance of infection by interfering with the assembly immune response, like modulation of cytokine production and non-activation of effectors cells. In the present work, we investigated whether LPGs from two *L*. *amazonensis* strains would account for differences in the interaction with macrophages and *L*. *migonei*.

LPG polymorphisms are common in the composition of branching sugars attached to the conserved repeat units of its backbone. While in the Old World species, a wide spectrum of sugar composition and structure is commonly observed, in New World species only glucose residues in the side chains of *Leishmania* were documented to date [17,21,23,24,43]. Our preliminary characterization of the repeat units using specific antibodies suggested the existence of intraspecies polymorphism in *L*. *amazonensis* LPGs with differences in the side-chains and in the level of glycosylation. The LPG of PH8 strain strongly reacted with CA7AE, that recognizes the basic backbone of the repeat units is Gal(β)Man-PO_4_ [21,34]. However, Josefa LPG did not reacted with this antibody, thus suggesting the existence of sugars as side-chains in the repeat units. This feature is commonly found in the LPG of *L*. *major* reference strain FV1, which does not react with CA7AE [17]. In order to evaluate the quality of the sugars branching-off the repeat units, LT22 and WIC.79.3 antibodies were used to detect the presence of glucose and galactose, respectively [21,35]. Based on *L*. *major* LPGs used as controls, they were either recognized by those antibodies, suggesting cross-reactivity. Moreover, those data reinforced the presence of either glucoses or galactoses as side-chains in *L*. *amazonensis* LPGs. A fully detailed biochemical analysis must await the results of further investigations.

Understanding variations and the LPG structures are crucial for the comprehension of the mechanisms of how parasites survive under extremely adverse conditions. Although the role of LPG in the interaction with the vertebrate host immune system has been studied, it is still unclear how its polymorphism affects the parasite survival. *L*. *amazonensis* LPG induces release of NETs and LTB4 production by neutrophils, thus contributing to diminish parasite burden in the *Leishmania* inoculation site [14,16]. Additionally, *L*. *mexicana* LPG induce TNF-α and IL-10 in monocytes, modulates IL-12 production and diminishes NF-κB nuclear translocation [44]. Here we show that LPGs from both *L*. *amazonensis* strains stimulates NO and cytokine production (TNF-α and IL-6) by peritoneal murine macrophages via TLR4. A similar cytokine production was also observed for other species such as *L*. *braziliensis* LPG, another important dermotropic species. However, this activation was primarily via TLR2 [30]. The NO production by macrophages play a central role in determining intracellular killing of *Leishmania* [45] and the intact structure of LPG appears to be important for this activation [12,29]. In many models, NO synthesis is dependent on a combination of IFN-γ and TNF-α via TLR-dependent mechanisms as an important leishmanicidal effector complex to macrophages [46]. In conclusion, the preliminary variations in the sugar motifs of LPG, did not result in any difference in macrophage activation/signaling thus suggesting the role of conserved motifs such as the lipid anchor [29].

Previous studies have demonstrated that different macrophage receptors mediate the uptake and phagocytosis of *Leishmania*. The early recognition of pathogens by cells capable of synthesizing cytokines is crucial for the adequate control of intracellular pathogens. Gene knockout studies in mice have suggested that TLR signaling is essential for the immune response against *Leishmania* parasites. Moreover, *Leishmania* LPGs and GIPLs are agonists of TLR2 and TLR4 [28–30,41,42]. Glyconjugates can modulate the host immune response and their activity seems to be structure dependent. The *L*. *braziliensis* LPG exerts a pro-inflammatory interaction with TLR2, inducing the production of NO and cytokines (IL-1β, TNF-α and IL-6). On the other hand, the *L*. *infantum* LPG was shown to be immunosuppressive and did not induce NO, cytokines and NF-κB translocation [30]. Our results indicate that LPG from both *L*. *amazonensis* strains induce the production of NO and cytokines in IFN-γ-primed macrophages via TLR4. However in other members of the *L*. *mexicana* complex, *L*. *mexicana* LPG activates either TLR2 or TLR4 leading to ERK and p38 MAPK phosphorylation and production of cytokines in human macrophages [42].

Thus, although it has been shown that LPG of *Leishmania* activates TLRs and that the engagement of these receptors is important for the infection, the complete intracellular processes that are involved in this activation remain unknown. Here we bring some light into the effects of LPG on MAPK and NF-κB signaling, a kinase and transcription factor known for their crucial role in immune defense against pathogens [44,47–49]. According to previous reports, infection by *L*. *amazonensis* altered phosphorylation of ERK1/2 in response to LPS in murine macrophages [50] and also activates a transcriptional repressor of the NF-κB [48,51]. Consistent with those observations, here LPGs from both *L*. *amazonensis* strains also activated p-IκBα, a NF-κB translocation inhibitor, via TLR4. Since no further NF-κB translocation was detected in the CHO cells, a possible mechanism that has been suggested favors its inhibition by p50/p50 NF-κB homodimer [55]. Moreover, *L*. *donovani* and *L*. *major* infection caused inactivation of ERK1/2 and p38, respectively, which was accompanied by the inhibition of transcription factors also modulation of cytokine production [52,53]. In contrast to GIPLs (with fail to activate MAPKs) [41], our data show that LPG from both *L*. *amazonensis* strains is equally activating MAPKs (p38 and JNK) and p-IκBα in peritoneal murine macrophages via TLR4 (Fig 3). On the other hand, these LPGs do not activate the NF-κB translocation. These and our results strongly suggest that *Leishmania* species have distinct mechanism of modulating the signaling pathways during immunopathological events.

The role of LPG during the interaction with the invertebrate host is a very controversial subject and it has been extensively investigated using *in vitro* and *in vivo* models [8,21,24,54,55]. Although the *in vitro* system has limitations [56], this model provided important evidence for parasite attachment in the sand fly midgut using many restricted and specific vector as classified elsewhere [57,58]. For example, successful binding to the midgut was reported using the Old World pairs *L*. *major/Phlebotomus papatasi* [8,54], *L*. *major/Phlebotomus duboscqi* [59] and *L*. *tropica/Phlebotomus sergenti* [60]. Perhaps, due its similarity to *L*. *major* LPG, who also possesses terminal β-galactosyl residues, *L*. *turanica* LPG may also be important for development in *P*. *papatasi* [61,62]. Moreover, the role of LPG has been questioned in permissive vectors such as *Lutzomyia longipalpis* and *Phlebotomus perniciosus*, where LPG mutants of *L*. *mexicana* and *L*. *major* were able to sustain infection in those vectors [63]. Recently, an alternative mechanism was suggested that flagellar protein FLAG1/SMP1 has been also implicated as an attachment binding candidate for specific and restricted vectors. In this work, a competitive binding assays using an antibody against FLAG1/SMP1 inhibited interaction using the pair *L*. *major* and *P*. *papatasi*. However, no effect was observed for permissive *L*. *longipalpis* [64].

The significance of LPG modifications was investigated during *in vivo* interaction of *L*. *amazonensis* with *L*. *migonei*. Although *L*. *amazonensis* is naturally transmitted by *L*. *flaviscutellata*, the absence of a colony led us to use an alternative sand fly, which had been previously shown to successfully harbor this parasite and *L*. *braziliensis* [5]. Since this species, although suspected, is not yet considered a natural proven vector of *L*. *amazonensis*, a high parasite doses was artificially offered to the sand flies. In spite of a loss after the 3^rd^ day, parasite multiplication inside the alimentary tract of the *L*. *migonei* was successful for both *L*. *amazonensis* strains. To survive, the parasites need avoid a number of barriers including the lethal effects of digestive enzymes in the early blood-fed midgut and the excretion with the digested blood meal [5,7,65,66]. The strong correlation between the excretion of blood meal and the sudden loss of promastigotes suggests that the inability of *Leishmania* strains to persist in an inappropriate sand fly is related to their failure to remain anchored to the gut wall via specific attachment sites [22,67]. Nevertheless, *L*. *migonei* was able to sustain infection with both of the *L*. *amazonensis* strains tested, regardless of the type of LPG. It seems likely that *L*. *migonei* together with *L*. *longipalpis* might be considered a permissive vector as previously suggested [57,58,68]. However, the fully development of those two *L*. *amazonensis* strains should be further investigated.

Some studies have determined that polymorphisms in the phosphoglycan domains of LPG might be crucial for *Leishmania* promastigotes to attach to the midgut and to maintain vector infection after blood meal excretion [9]. Additional support is based on the altered behavior of LPG deficient *L*. *donovani* and *L*. *major* mutant promastigotes (lpg-) who showed diminished capacity to maintain infection within the sand fly midgut [54,69]. Furthermore, it was recently presented the occurrence of intraspecies polymorphism in *L*. *infantum* LPG. Also, the biological role of the three LPG types (I, II and III) was studied during the interaction with the vector *L*. *longipalpis* [24]. Consistent with our results, all strains could successfully sustain infection in this vector, indicating that LPG polymorphisms did not affect this process. In spite of having a strong evidence for the existence of a midgut receptor for LPG, there is no current information in *L*. *migonei*. Indeed, the only known receptor was described for *L*. *major*, a galectin receptor found in the midgur of *P*. *papatasi* binding to LPG β-galactose residues [9,70]. The existence of midgut glycoproteins bearing terminal N-acetylgalactosamine in sand fly was also suggested as a putative parasite ligand [71].

Here we describe for the first time the immunomodulary properties of two LPGs isolated from different hosts. Those LPGs were equally able to trigger NO and cytokine (TNF-α and IL-6) production via TLR4. The preliminary differences in carbohydrate structure did not seem to affect the interaction of these strains with macrophages and the sand fly vector.

## Supporting Information

S1 Fig Growth curves of *L*. *amazonensis*. (A) *L*. *amazonensis* (PH8 and Josefa strains) were grown in M199 medium and counts determined daily (initial concentration of 1 × 10^5^/mL). (B) Restriction fragment length polymorphisms of 120 bp kDNA amplicons from *Leishmania* obtained with restriction enzyme Hae III and analyzed on silver-stained 10% polyacrylamide gel. MM: 50 bp molecular size marker; lanes: Lb–*L*. *braziliensis* (MHOM/BR/75/M2903), Li–*L*. *infantum* (MHOM/BR/74/PP75); La–*L*. *amazonensis* reference (IFLA/BR/67/PH8), PH8 –*L*. *amazonensis* PH8 (IFLA/BR/67/PH8) and Jos–*L*. *amazonensis* Josefa (MHOM/BR/75/Josefa).
(TIF)

S2 Fig Dot-blots of *Leishmania* LPGs using different mAb antibodies. Purified LPGs from *L*. *amazonensis* strains (PH8 and Josefa), *L*. *infantum* (BH46 strain) and *L*. *major* strains (FV1 and LV39) were probed with the mAbs CA7AE (1:1000), LT22 (1:1000) and WIC 79.3 (1:1000). Peroxidase-conjugated anti-mouse IgG (1:5000) was used as secondary antibody. The reaction was developed with luminol.
(TIF)

S3 Fig Cytokine production by IFN-γ primed macrophages stimulated with LPG and live parasites. Cells were pre-incubated with IFN-γ (3 IU/mL) for the 18 h then 10 μg/mL of LPG, and supernatants used for cytokine IL-10 (A), IL-1β (B) and IL-12 (C) measurements were collected 48 h latter. Fresh medium alone was used as negative control cells and LPS (100 ng/mL) as a positive control. Cytokine concentrations were determined by flow cytometry. C = negative control; LPG PH8 = *L*. *amazonensis* LPG PH8 strain; LPG Jos = *L*. *amazonensis* LPG Josefa strain; La PH8 = *L*. *amazonensis* PH8 live promastigotes and La Jos = *L*. *amazonensis* Josefa live promastigotes. Results represent the mean ± SD of 3 experiments in duplicate, * = P< 0.05 was considered significant.
(TIF)

## References

[1] Herwaldt BL. Leishmaniasis. Lancet. 1999;354: 1191–1199. 10513726 10.1016/S0140-6736(98)10178-2

[2] Silveira FT, Lainson R, De Castro Gomes CM, Laurenti MD, Corbett CEP. Immunopathogenic competences of *Leishmania (V*.*) braziliensis* and *L*. *(L*.*) amazonensis* in American cutaneous leishmaniasis. Parasite Immunol. 2009;31: 423–431. 10.1111/j.1365-3024.2009.01116.x 19646206

[3] Carvalho BM, Rangel EF, Ready PD, Vale MM. Ecological Niche Modelling Predicts Southward Expansion of *Lutzomyia (Nyssomyia) flaviscutellata* (Diptera: Psychodidae: Phlebotominae), Vector of *Leishmania (Leishmania) amazonensis* in South America, under Climate Change. PLoS ONE. 2015;10(11). 10.1371/journal.pone.0143282 PMC4664266 26619186

[4] Grimaldi G, Tesh RB, McMahon-Pratt D. A review of the geographic distribution and epidemiology of leishmaniasis in the New World. American Journal of Tropical Medicine and Hygiene. 1989 pp. 687–725. 2701633 10.4269/ajtmh.1989.41.687

[5] Nieves E, Pimenta PFP. Development of *Leishmania (Viannia) braziliensis* and *Leishmania (Leishmania) amazonensis* in the sand fly *Lutzomyia migonei* (Diptera: Psychodidae). 2000;37: 134–140. 10.1603/0022-2585-37.1.134 15218917

[6] Lainson R. Ecological interactions in the transmission of the leishmaniases. Philos Trans R Soc Lond B Biol Sci. 1988;321: 389–404. 2907150 10.1098/rstb.1988.0099

[7] De Assis RR, Ibraim IC, Nogueira PM, Soares RP, Turco SJ. Glycoconjugates in New World species of *Leishmania*: Polymorphisms in lipophosphoglycan and glycoinositolphospholipids and interaction with hosts. Biochim Biophys Acta. Elsevier B.V.; 2012;1820: 1354–1365. 10.1016/j.bbagen.2011.11.001 22093608

[8] Pimenta PF, Saraiva EM, Rowton E, Modi GB, Garraway L a, Beverley SM, et al Evidence that the vectorial competence of phlebotomine sand flies for different species of *Leishmania* is controlled by structural polymorphisms in the surface lipophosphoglycan. Proc Natl Acad Sci U S A. 1994;91: 9155–9159. 8090785 10.1073/pnas.91.19.9155 PMC44766

[9] Kamhawi S, Ramalho-Ortigao M, Van MP, Kumar S, Lawyer PG, Turco SJ, et al A role for insect galectins in parasite survival. Cell. 2004;119: 329–341. 15543683 10.1016/j.cell.2004.10.009

[10] Brittingham A, Mosser D. Exploitation of the complement system by *Leishmania* promastigotes. Parasitol Today. 1996;12: 444–447. 15275279 10.1016/0169-4758(96)10067-3

[11] Späth GF, Garraway L a, Turco SJ, Beverley SM. The role(s) of lipophosphoglycan (LPG) in the establishment of *Leishmania major* infections in mammalian hosts. Proc Natl Acad Sci U S A. 2003;100: 9536–9541. 12869694 10.1073/pnas.1530604100 PMC170953

[12] Proudfoot L, Nikolaev a V, Feng GJ, Wei WQ, Ferguson M a, Brimacombe JS, et al Regulation of the expression of nitric oxide synthase and leishmanicidal activity by glycoconjugates of *Leishmania* lipophosphoglycan in murine macrophages. Proc Natl Acad Sci U S A. 1996;93: 10984–10989. 8855295 10.1073/pnas.93.20.10984 PMC38270

[13] Giorgione JR, Turco SJ, Epand RM. Transbilayer inhibition of protein kinase C by the lipophosphoglycan from *Leishmania donovani*. Proc Natl Acad Sci U S A. 1996;93: 11634–11639. 8876188 10.1073/pnas.93.21.11634 PMC38110

[14] Guimarães-Costa AB, Nascimento MTC, Froment GS, Soares RPP, Morgado FN, Conceição-Silva F, et al *Leishmania amazonensis* promastigotes induce and are killed by neutrophil extracellular traps. Proc Natl Acad Sci U S A. 2009;106: 6748–6753. 10.1073/pnas.0900226106 19346483 PMC2672475

[15] de Carvalho Vivarini a., Pereira RDMS, Dias Teixeira KL, Calegari-Silva TC, Bellio M, Laurenti MD, et al Human cutaneous leishmaniasis: interferon-dependent expression of double-stranded RNA-dependent protein kinase (PKR) via TLR2. FASEB J. 2011;25: 4162–4173. 10.1096/fj.11-185165 21846836

[16] Tavares NM, Araújo-Santos T, Afonso L, Nogueira PM, Lopes UG, Soares RP, et al Understanding the Mechanisms Controlling *Leishmania amazonensis* Infection In Vitro: The Role of LTB4 Derived From Human Neutrophils. J Infect Dis. 2014;210: 1–11. 24634497 10.1093/infdis/jiu158 PMC4111911

[17] McConville MJ, Turco SJ, Ferguson M a, Sacks DL. Developmental modification of lipophosphoglycan during the differentiation of *Leishmania major* promastigotes to an infectious stage. EMBO J. 1992;11: 3593–3600. 1396559 10.1002/j.1460-2075.1992.tb05443.x PMC556818

[18] McConville MJ, Schnur LF, Jaffe C, Schneider P. Structure of *Leishmania* lipophosphoglycan: inter- and intra-specific polymorphism in Old World species. Biochem J. 1995;310: 807–818. 7575413 10.1042/bj3100807 PMC1135969

[19] Sacks DL, Pimenta PF, McConville MJ, Schneider P, Turco SJ. Stage-specific binding of *Leishmania donovani* to the sand fly vector midgut is regulated by conformational changes in the abundant surface lipophosphoglycan. J Exp Med. 1995;181: 685–697. 7836922 10.1084/jem.181.2.685 PMC2191891

[20] Mahoney AB, Sacks DL, Saraiva E, Modi G, Turco SJ. Intra-species and stage-specific polymorphisms in lipophosphoglycan structure control *Leishmania donovani*—Sand fly interactions. Biochemistry. 1999;38: 9813–9823. 10433687 10.1021/bi990741g

[21] Soares RPP, Macedo ME, Ropert C, Gontijo NF, Almeida IC, Gazzinelli RT, et al *Leishmania chagasi*: Lipophosphoglycan characterization and binding to the midgut of the sand fly vector *Lutzomyia longipalpis*. Mol Biochem Parasitol. 2002;121: 213–224. 12034455 10.1016/s0166-6851(02)00033-6

[22] Soares RPP, Barron T, McCoy-Simandle K, Svobodova M, Warburg A, Turco SJ. *Leishmania tropica*: Intraspecific polymorphisms in lipophosphoglycan correlate with transmission by different *Phlebotomus* species. Exp Parasitol. 2004;107: 105–114. 15208044 10.1016/j.exppara.2004.05.001

[23] Soares RPP, Cardoso TL, Barron T, Araújo MSS, Pimenta PFP, Turco SJ. *Leishmania braziliensis*: A novel mechanism in the lipophosphoglycan regulation during metacyclogenesis. Int J Parasitol. 2005;35: 245–253. 15722076 10.1016/j.ijpara.2004.12.008

[24] Coelho-Finamore JM, Freitas VC, Assis RR, Melo MN, Novozhilova N, Secundino NF, et al *Leishmania infantum*: Lipophosphoglycan intraspecific variation and interaction with vertebrate and invertebrate hosts. Int J Parasitol. Australian Society for Parasitology Inc.; 2011;41: 333–342. 10.1016/j.ijpara.2010.10.004 21118695

[25] Paranaíba L, de Assis R, Nogueira P, Torrecilhas A, Campos J, Silveira A, et al *Leishmania enriettii*: biochemical characterisation of lipophosphoglycans (LPGs) and glycoinositolphospholipids (GIPLs) and infectivity to *Cavia porcellus*. Parasit Vectors. 2015;8: 31 10.1186/s13071-015-0633-8 25595203 PMC4311450

[26] Passero LFD, Assis RR, da Silva TNF, Nogueira PM, Macedo DH, Pessoa NL, et al Differential modulation of macrophage response elicited by glycoinositolphospholipids and lipophosphoglycan from *Leishmania (Viannia) shawi*. Parasitol Int. Elsevier Ireland Ltd; 2015;64: 32–35. 10.1016/j.parint.2015.01.006 25619843

[27] Turco SJ, Descoteaux A. The Lipophosphoglycan of *Leishmania* Parasites. 1992; 65–94. 10.1146/annurev.mi.46.100192.000433 1444269

[28] Becker I, Salaiza N, Aguirre M, Delgado J, Carrillo-Carrasco N, Kobeh LG, et al *Leishmania* lipophosphoglycan (LPG) activates NK cells through toll-like receptor-2. Mol Biochem Parasitol. 2003;130: 65–74. 12946842 10.1016/s0166-6851(03)00160-9

[29] de Veer MJ, Curtis JM, Baldwin TM, DiDonato J a., Sexton A, McConville MJ, et al MyD88 is essential for clearance of *Leishmania major*: Possible role for lipophosphoglycan and Toll-like receptor 2 signaling. Eur J Immunol. 2003;33: 2822–2831. 14515266 10.1002/eji.200324128

[30] Ibraim IC, de Assis RR, Pessoa NL, Campos MA, Melo MN, Turco SJ, et al Two biochemically distinct lipophosphoglycans from *Leishmania braziliensis* and *Leishmania infantum* trigger different innate immune responses in murine macrophages. Parasit Vectors. 2013;6: 54 10.1186/1756-3305-6-54 23497381 PMC3606350

[31] Garcia L, Kindt A, Bermudez H, Llanos-Cuentas A, De Doncker S, Arevalo J, et al Culture-Independent Species Typing of Neotropical *Leishmania* for Clinical Validation of a PCR-Based Assay Targeting Heat Shock Protein 70 Genes. J Clin Microbiol. 2004;42: 2294–2297. 15131217 10.1128/JCM.42.5.2294-2297.2004 PMC404633

[32] Orlandi PA, Turco SJ. Structure of the lipid moiety of the *Leishmania donovani* lipophosphoglycan. J Biol Chem. 1987;262: 10384–10391. 3611065

[33] Dubois M, Gilles KA, Hamilton JKJ, Rebers PA, Smith F. Colorimetric method for determination of sugars and related substances. Anal Chem. 1956;28: 350–356.

[34] Tolson DL, Turco SJ, Beecroft RP, Pearson TW. The immunochemical structure and surface arrangement of *Leishmania donovani* lipophosphoglycan determined using monoclonal antibodies. Mol Biochem Parasitol. 1989;35: 109–118. 2475775 10.1016/0166-6851(89)90113-8

[35] Kelleher M, Curtis JM, Sacks DL, Handman E, Bacic A. Epitope mapping of monoclonal antibodies directed against lipophosphoglycan of *Leishmania major* promastigotes. Mol Biochem Parasitol. 1994;66: 187–200. 7808469 10.1016/0166-6851(94)90146-5

[36] Kolodziej H, Radtke O a., Kiderlen AF. Stimulus (polyphenol, IFN-y, LPS)-dependent nitric oxide production and antileishmanial effects in RAW 264.7 macrophages. Phytochemistry. Elsevier Ltd; 2008;69: 3103–3110. 10.1016/j.phytochem.2007.11.012 18164321

[37] Shapiro SS. JSTOR: Biometrika, Vol. 52, No. 3/4 (Dec., 1965), pp. 591–611. Biometrika. 1965;

[38] Desjeux P. Leishmaniasis: Current situation and new perspectives. Comp Immunol Microbiol Infect Dis. 2004;27: 305–318. 15225981 10.1016/j.cimid.2004.03.004

[39] Silveira FT, Lainson R, Corbett CEP. Further observations on clinical, histopathological, and immunological features of borderline disseminated cutaneous leishmaniasis caused by *Leishmania (Leishmania) amazonensis*. Mem Inst Oswaldo Cruz. 2005;100: 525–534. 16184231 10.1590/s0074-02762005000500013

[40] Sacks D, Kamhawi S. Molecular Aspects of Parasite-Vector and Vector-Host Interacions in Leishmaniasis. 2001; 10.1146/annurev.micro.55.1.453 11544364

[41] Assis RR, Ibraim IC, Noronha FS, Turco SJ, Soares RP. Glycoinositolphospholipids from *Leishmania braziliensis* and *Leishmania infantum*: Modulation of innate immune system and variations in carbohydrate structure. PLoS Negl Trop Dis. 2012;6: 1–11. 10.1371/journal.pntd.0001543 PMC3289616 22389743

[42] Rojas-Bernabé A, Garcia-Hernández O, Maldonado-Bernal C, Delegado-Dominguez J, Ortega E, Gutiérrez-Kobeh L, et al *Leishmania mexicana* lipophosphoglycan activates ERK and p38 MAP kinase and induces production of proinflammatory cytokines in human macrophages through TLR2 and TLR4. Parasitology. 2014;141: 788–800. 10.1017/S0031182013002187 24512642

[43] Ilg T, Etgess R, Overath P, Mcconvilleq MJ, Thomas-oatest J, Thomasll J, et al Structure of *Leishmania mexicana* Lipophosphoglycan. 1992; 1551890

[44] Argueta-Donohué J, Carrillo N, Valdés-Reyes L, Zentella A, Aguirre-García M, Becker I, et al *Leishmania mexicana*: Participation of NF-κB in the differential production of IL-12 in dendritic cells and monocytes induced by lipophosphoglycan (LPG). Exp Parasitol. 2008;120: 1–9. 10.1016/j.exppara.2008.04.002 18508052

[45] Holzmuller P, Bras-Gonçalves R, Lemesre J-L. Phenotypical characteristics, biochemical pathways, molecular targets and putative role of nitric oxide-mediated programmed cell death in *Leishmania*. Parasitology. 2006;132 Suppl: S19–S32. 17018162 10.1017/S0031182006000837

[46] Mosser DM, Edwards JP. Exploring the full spectrum of macrophage activation. Nat Rev Immunol. 2008;8: 958–969. 10.1038/nri2448 19029990 PMC2724991

[47] Cameron P, McGachy A, Anderson M, Paul A, Coombs GH, Mottram JC, et al Inhibition of lipopolysaccharide-induced macrophage IL-12 production by *Leishmania mexicana* amastigotes: the role of cysteine peptidases and the NF-kappaB signaling pathway. J Immunol. 2004;173: 3297–3304. 15322192 10.4049/jimmunol.173.5.3297

[48] Calegari-Silva TC, Pereira RMS, De-Melo LDB, Saraiva EM, Soares DC, Bellio M, et al NF-κB-mediated repression of iNOS expression in *Leishmania amazonensis* macrophage infection. Immunol Lett. 2009;127: 19–26. 10.1016/j.imlet.2009.08.009 19712696

[49] Yang Z, Mosser DM, Zhang X. Activation of the MAPK, ERK, following *Leishmania amazonensis* infection of macrophages. J Immunol. 2007;178: 1077–1085. 17202371 10.4049/jimmunol.178.2.1077 PMC2643020

[50] Martiny A, Meyer-Fernandes JR, De Souza W, Vannier-Santos MA. Altered tyrosine phosphorylation of ERK1 MAP kinase and other macrophage molecules caused by *Leishmania* amastigotes. Mol Biochem Parasitol. 1999;102: 1–12. 10477171 10.1016/s0166-6851(99)00067-5

[51] Calegari-silva TC, Vivarini C, Miqueline M, Dos Santos GRRM, Teixeira KL, Saliba AM, et al The human parasite *Leishmania amazonensis* downregulates iNOS expression via NF- k B p50 / p50 homodimer : role of the PI3K / Akt pathway. Open Biol. 2015;5. 10.1098/rsob.150118 PMC4593669 26400473

[52] Srivastava A, Singh N, Mishra M, Kumar V, Gour JK, Bajpai S, et al Identification of TLR inducing Th1-responsive *Leishmania donovani* amastigote-specific antigens. Mol Cell Biochem. 2012;359: 359–368. 10.1007/s11010-011-1029-5 21858498

[53] Sarkar A, Aga E, Bussmeyer U, Bhattacharyya A, Möller S, Hellberg L, et al Infection of neutrophil granulocytes with *Leishmania major* activates ERK 1/2 and modulates multiple apoptotic pathways to inhibit apoptosis. Med Microbiol Immunol. 2013;202: 25–35. 10.1007/s00430-012-0246-1 22661217

[54] Pimenta PF, Turco SJ, McConville MJ, Lawyer PG, Perkins P V, Sacks DL. Stage-specific adhesion of *Leishmania* promastigotes to the sandfly midgut. Science. 1992;256: 1812–1815. 1615326 10.1126/science.1615326

[55] Soares RP, Margonari C, Secundino NC, MacÊdo ME, Da Costa SM, Rangel EF, et al Differential midgut attachment of *Leishmania (Viannia) braziliensis* in the sand flies *Lutzomyia (Nyssomyia) whitmani* and *Lutzomyia (Nyssomyia) intermedia*. J Biomed Biotechnol. 2010;2010 10.1155/2010/439174 PMC2789580 20011070

[56] Wilson R, Bates MD, Dostalova A, Jecna L, Dillon RJ, Volf P, et al Stage-Specific Adhesion of Leishmania Promastigotes to Sand Fly Midguts Assessed Using an Improved Comparative Binding Assay. PLoS Negl Trop Dis. 2010;4: e816 10.1371/journal.pntd.0000816 20838647 PMC2935393

[57] Kamhawi S. Phlebotomine sand flies and *Leishmania* parasites: friends or foes? Trends Parasitol. 2006;22: 439–445. 16843727 10.1016/j.pt.2006.06.012

[58] Volf P, Myskova J. Sand flies and *Leishmania*: specific versus permissive vectors. Trends Parasitol. 2007;23: 91–92. 17207663 10.1016/j.pt.2006.12.010 PMC2839922

[59] Svárovská A, Ant TH, Seblová V, Jecná L, Beverley SM, Volf P. *Leishmania major* glycosylation mutants require phosphoglycans (lpg2 -) but not lipophosphoglycan (lpg1-) for survival in permissive sand fly vectors. PLoS Negl Trop Dis. 2010;4: 1–7. 10.1371/journal.pntd.0000580 PMC2797086 20084096

[60] Kamhawi S, Modi GB, Pimenta PF, Rowton E, Sacks DL. The vectorial competence of *Phlebotomus sergenti* is specific for *Leishmania tropica* and is controlled by species-specific, lipophosphoglycan-mediated midgut attachment. Parasitology. 2000;121 (Pt 1: 25–33. 11085222 10.1017/s0031182099006125

[61] Chajbullinova A, Votypka J, Sadlova J, Kvapilova K, Seblova V, Kreisinger J, et al The development of *Leishmania turanica* in sand flies and competition with *L*. *major*. Parasit Vectors. Parasites & Vectors; 2012;5: 219 10.1186/1756-3305-5-219 23031344 PMC3484061

[62] Volf P, Nogueira PM, Myskova J, Turco SJ, Soares RP. Structural comparison of lipophosphoglycan from *Leishmania turanica* and *L*. *major*, two species transmitted by *Phlebotomus papatasi*. Parasitol Int. Elsevier Ireland Ltd; 2014;63: 683–686. 10.1016/j.parint.2014.05.004 24863491

[63] Jecna L, Dostalova A, Wilson R, Seblova V, Chang K-P, Bates P a, et al The role of surface glycoconjugates in *Leishmania* midgut attachment examined by competitive binding assays and experimental development in sand flies. Parasitology. 2013;140: 1026–32. 10.1017/S0031182013000358 23611086

[64] Di-Blasi T, Lobo AR, Nascimento LM, Córdova-Rojas JL, Pestana K, Marín-Villa M, et al The Flagellar Protein FLAG1/SMP1 is a Candidate for *Leishmania*–Sand Fly Interaction. Vector-Borne Zoonotic Dis. 2015;15: 202–209. 10.1089/vbz.2014.1736 25793476 PMC4939454

[65] Sacks DL. *Leishmania*-sand fly interactions controlling species-specific vector competence. Cell Microbiol. 2001;3: 189–196. 11298643 10.1046/j.1462-5822.2001.00115.x

[66] Dostálová A, Volf P. *Leishmania* development in sand flies: parasite-vector interactions overview. Parasit Vectors. 2012;5: 276 10.1186/1756-3305-5-276 23206339 PMC3533922

[67] Soares RP, Turco SJ. *Lutzomyia longipalpis* (Diptera: Psychodidae: Phlebotominae): a review. An Acad Bras Cienc. 2003;75: 301–330. 12947480 10.1590/s0001-37652003000300005

[68] Guimarães VCFV, Pruzinova K, Sadlova J, Volfova V, Myskova J, Filho SPB, et al *Lutzomyia migonei* is a permissive vector competent for *Leishmania infantum*. Parasit Vectors. Parasites & Vectors; 2016;9: 159 10.1186/s13071-016-1444-2 26988559 PMC4797322

[69] Butcher BA, Turco SJ, Hilty BA, Pimenta PF, Panunzio M, Sacks DL. Deficiency in b1, 3-Galactosyltransferase of a *Leishmania major* Lipophosphoglycan Mutant Adversely Influences the *Leishmania* -Sand Fly Interaction. 1996;271: 20573–20579. 10.1074/jbc.271.34.20573 8702802

[70] Dillon RJ, Lane RP. Detection of *Leishmania* lipophosphoglycan binding proteins in the gut of the sandfly vector. 1999; 27–32. 10.1017/s0031182098003588 10070658

[71] Myskova J, Svobodova M, Beverley SM, Volf P. A lipophosphoglycan-independent development of *Leishmania* in permissive sand flies. Microbes Infect. 2007;9: 317–324. 17307009 10.1016/j.micinf.2006.12.010 PMC2839925

