# Supplementary figures and images for: Lipophosphoglycans from Leishmania amazonensis Strains Display Immunomodulatory Properties via TLR4 and Do Not Affect Sand Fly Infection
Source: PLoS Negl Trop Dis. 2016 Aug 10;10(8):e0004848. doi: 10.1371/journal.pntd.0004848 (PMC4980043; doi:10.1371/journal.pntd.0004848)

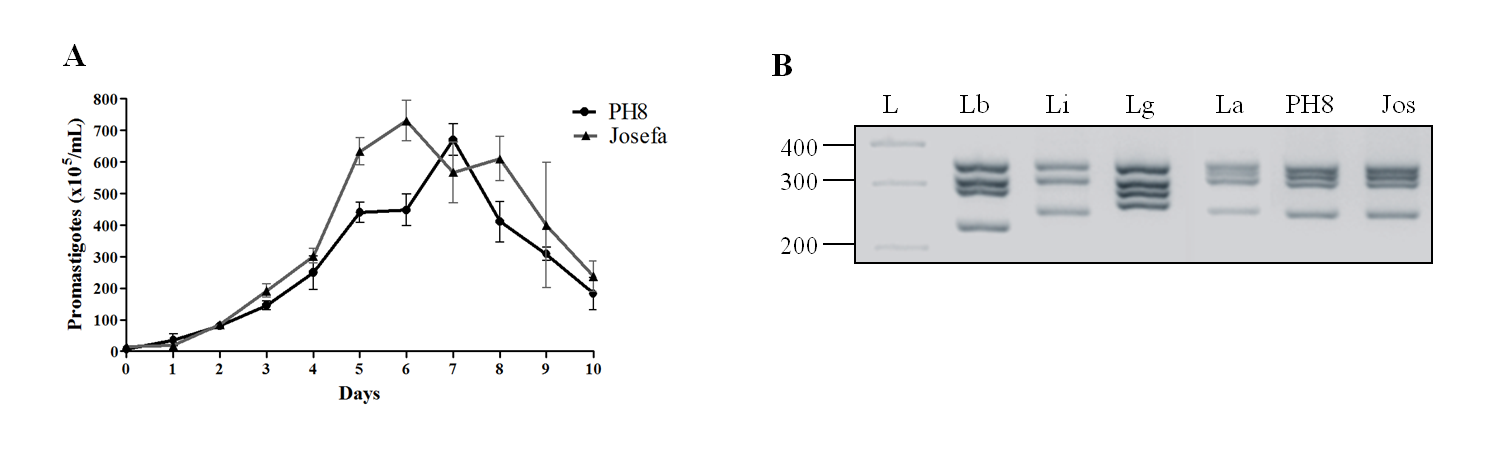

Supplement: S1 Fig — (A) L. amazonensis (PH8 and Josefa strains) were grown in M199 medium and counts determined daily (initial concentration of 1 × 10^5^/mL). (B) Restriction fragment length polymorphisms of 120 bp kDNA amplicons from Leishmania obtained with restriction enzyme Hae III and analyzed on silver-stained 10% polyacrylamide gel. MM: 50 bp molecular size marker; lanes: Lb–L. braziliensis (MHOM/BR/75/M2903), Li–L. infantum (MHOM/BR/74/PP75); La–L. amazonensis reference (IFLA/BR/67/PH8), PH8 –L. amazonensis PH8 (IFLA/BR/67/PH8) and Jos–L. amazonensis Josefa (MHOM/BR/75/Josefa). (TIF) [file pntd.0004848.s001.tif]

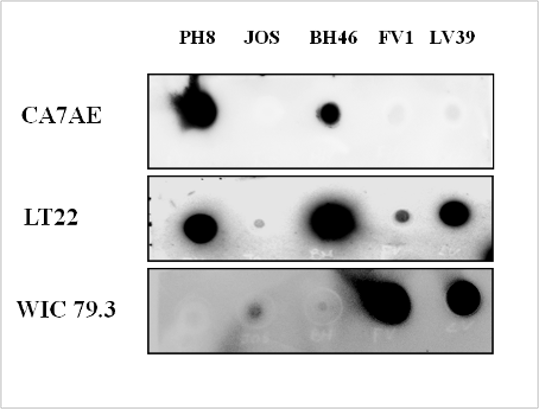

Supplement: S2 Fig — Purified LPGs from L. amazonensis strains (PH8 and Josefa), L. infantum (BH46 strain) and L. major strains (FV1 and LV39) were probed with the mAbs CA7AE (1:1000), LT22 (1:1000) and WIC 79.3 (1:1000). Peroxidase-conjugated anti-mouse IgG (1:5000) was used as secondary antibody. The reaction was developed with luminol. (TIF) [file pntd.0004848.s002.tif]

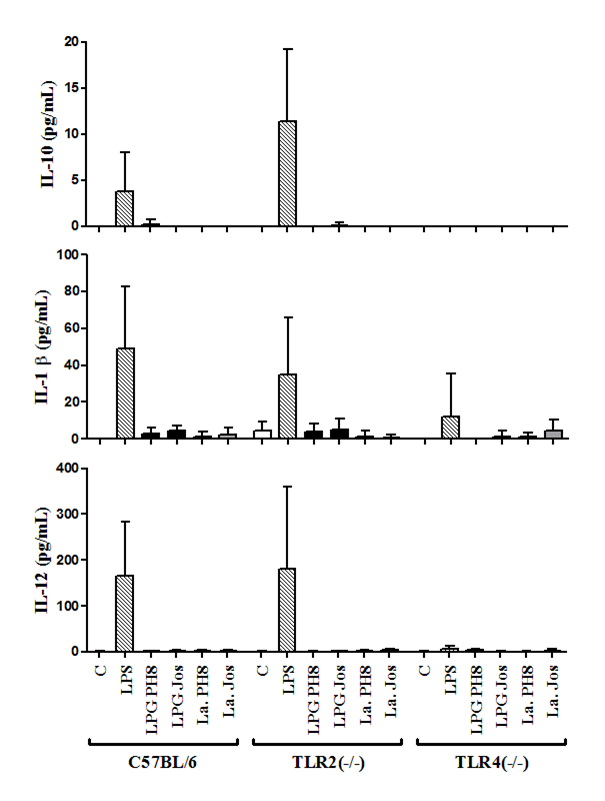

Supplement: S3 Fig — Cells were pre-incubated with IFN-γ (3 IU/mL) for the 18 h then 10 μg/mL of LPG, and supernatants used for cytokine IL-10 (A), IL-1β (B) and IL-12 (C) measurements were collected 48 h latter. Fresh medium alone was used as negative control cells and LPS (100 ng/mL) as a positive control. Cytokine concentrations were determined by flow cytometry. C = negative control; LPG PH8 = L. amazonensis LPG PH8 strain; LPG Jos = L. amazonensis LPG Josefa strain; La PH8 = L. amazonensis PH8 live promastigotes and La Jos = L. amazonensis Josefa live promastigotes. Results represent the mean ± SD of 3 experiments in duplicate, * = P< 0.05 was considered significant. (TIF) [file pntd.0004848.s003.tif]
